# MultiPSQ: A Software Solution for the Analysis of Diagnostic n-Plexed Pyrosequencing Reactions

**DOI:** 10.1371/journal.pone.0060055

**Published:** 2013-03-26

**Authors:** Piotr Wojtek Dabrowski, Kati Schröder, Andreas Nitsche

**Affiliations:** 1 Central Administration 4 (IT), Robert Koch Institute, Berlin, Germany; 2 Centre for Biological Threats and Special Pathogens 1, Robert Koch Institute, Berlin, Germany; University of Guelph, Canada

## Abstract

**Background:**

Pyrosequencing can be applied for Single-Nucleotide-Polymorphism (SNP)-based pathogen typing or for providing sequence information of short DNA stretches. However, for some pathogens molecular typing cannot be performed relying on a single SNP or short sequence stretch, necessitating the consideration of several genomic regions. A promising rapid approach is the simultaneous application of multiple sequencing primers, called multiplex pyrosequencing. These primers generate a fingerprint-pyrogram which is constituted by the sum of all individual pyrograms originating from each primer used.

**Methods:**

To improve pyrosequencing-based pathogen typing, we have developed the software tool MultiPSQ that expedites the analysis and evaluation of multiplex-pyrograms. As a proof of concept, a multiplex pyrosequencing assay for the typing of orthopoxviruses was developed to analyse clinical samples diagnosed in the German Consultant Laboratory for Poxviruses.

**Results:**

The software tool MultiPSQ enabled the analysis of multiplex-pyrograms originating from various pyrosequencing primers. Thus several target regions can be used for pathogen typing based on pyrosequencing. As shown with a proof of concept assay, SNPs present in different orthopoxvirus strains could be identified correctly with two primers by MultiPSQ.

**Conclusions:**

Software currently available is restricted to a fixed number of SNPs and sequencing primers, severely limiting the usefulness of this technique. In contrast, our novel software MultiPSQ allows analysis of data from multiplex pyrosequencing assays that contain any number of sequencing primers covering any number of polymorphisms.

## Introduction

One sensitive way to diagnose and subtype viral pathogens is usually based on the detection of viral nucleic acids via PCR amplification and subsequent DNA sequencing. For this purpose Sanger sequencing is commonly used to determine the viral species [1]. As a novel approach, pyrosequencing is a sequencing-by-synthesis method allowing a fast and reliable detection of single nucleotide polymorphisms (SNP) and sequence variations between different species [2]–[6]. The pyrosequencing reaction relies on the indirect detection of pyrophosphate that is released during sequence-specific nucleotide incorporation. Pyrophosphate is converted into discrete light signals proportional to the number of identical nucleotides incorporated. Light signals are detected by a charge-coupled device (CCD) camera and graphed in a so-called pyrogram [7].

**Figure 1:**
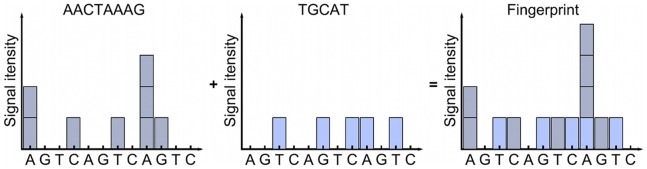
Principle of multiplex pyrosequencing. Several sequencing primers are used in a single reaction. The incorporation of nucleotides following each of the primers independently leads to the generation of light signals. These signals overlap in the final pyrogram, resulting in a characteristic fingerprint. Changes in any of the single sequences lead to a change in the fingerprint which allows classification based on SNPs from different regions of a PCR product or even from different PCR products.

In some cases the differentiation of viral types is not possible by detecting only one SNP or by considering only one sequence stretch as the same sequence variation can be found in different species. One approach to circumventing this problem is the use of a pool of type-specific primers [8], [9]. Another approach is the detection of multiple sequence variations in one region or in several regions in the same reaction by multiplex pyrosequencing with several sequencing primers [7], [10]–[12]. Here, one or more DNA templates containing relevant sequence variations are amplified by PCR using one biotinylated primer. Biotin-labelled ssDNA strands are separated, followed by hybridization of two or more sequencing primers. During pyrosequencing nucleotides are dispensed in a defined order and incorporated following each primer sequence. Since the signals from these simultaneous sequencing reactions are generated at the same time, they overlap to result in a single pyrogram (Figure 1). This pyrogram cannot be used directly for sequence analysis as it is unknown from which of the sequencing reactions the respective light signal originates. However, the resulting single pyrogram can be used as a unique fingerprint which is representative of a specific combination of sequences.

Using software currently commercially available, analysis of multiplex pyrograms is still challenging. Therefore, an in-house software tool called MultiPSQ was developed to analyse and evaluate multiplex pyrosequencing results. To verify this software, we developed a multiplex pyrosequencing assay identifying all human-pathogenic orthopoxviruses (OPV). In addition to the causative agent of smallpox, variola virus (VARV), the genus OPV comprises diverse species including those transmitted zoonotically and causing infections in humans, like monkeypox virus (MPXV), cowpox virus (CPXV) and vaccinia virus (VACV). Further OPV that do not infect humans are camelpox virus (CMLV), mousepox virus (ECTV), raccoonpox virus and taterapox virus as well as various unclassified OPV. As genomic sequences are well conserved among OPV, virus type differentiation by only sequence variations or SNPs within a single stretch of sequence is often impossible. This makes OPV a good candidate for the demonstration of the utility of multiplex pyrosequencing and our tool’s capabilities.

## Materials and Methods

### PCR with Biotinylated Primers

Each 25 µl reaction contained 3 µl of DNA, 1 × PCR buffer (Invitrogen), 4 mM of MgCl_2_, 200 nM of each dNTP, 800 nM of each PCR primer (mPox F: gTgATTTTggCAATAgTCCATgT, mPox R bio: Biotin-TAAAATggCCgAggAATTTg; Invitrogen) and 1 U of Platinum Taq polymerase (Invitrogen). PCR was performed in an Eppendorf Thermocycler (Eppendorf) with initial denaturation at 95°C for 5 min, followed by 45 cycles of 95°C for 15 s, 54°C for 30 s and 72°C for 30 s and a final elongation at 72°C for 5 min.

### Gelelectrophoresis and Quantification

To quantify amplified PCR product which represents the DNA template for pyrosequencing, 2 µl of PCR product were mixed with 1 µl of 6 × loading buffer and loaded on a 1.5% Agarose gel containing 0.5 µg/ml ethidium bromide. Equally, 2 µl of low mass DNA ladder (Invitrogen) were loaded in parallel. Electrophoresis was performed in 1 × TAE buffer at 90 V for 30 min. Quantification of DNA in resulting bands was done with E.A.S.Y RH-3 software (Herolab).

### Multiplex Pyrosequencing

Prior to sequencing, 20 to 25 µl of biotinylated PCR product were captured with Streptavidin Sepharose beads and purified with the PyroMark Q96 Vacuum Prep Workstation according to the manufacturer’s instructions (Qiagen). For pyrosequencing, 10 pmol of each sequencing primer (mPox PS1: AAATTCCggATACAT and mPox PS2: CTTgACAAAAATTgT) were hybridized to a single-stranded DNA template in the same well. Multiplex pyrosequencing using both primers at the same time for each template was performed with Pyro-Mark ID System (Qiagen) with programmed nucleotide dispensation order of eight cycles AGTC.

### Viral DNA from Cell Culture and Clinical Samples

The multiplex PSQ assay was established with viral DNA extracted from infected cell culture supernatants. DNA of the following viral strains was used: CMLV CP-19, CPXV GuWi, CPXV Brighton Red, CPXV Calpox, ECTV MP-Nü, MPXV MSF (the latter two kindly provided by Dr. H. Meyer, Bundeswehr Institute of Microbiology, Munich, Germany) and VACV NYCBH (ATCC # VR-1536).

To verify the multiplex PSQ assay established, DNA from 34 clinical samples provided by the German Consultant Laboratory for Poxviruses were analysed and differentiated with the novel approach. Using real-time PCR approaches, 31 out of these 34 samples had previously been diagnosed as OPV positive, two as parapoxvirus (PPV) positive and one as Molluscum contagiosum virus (MOCV) positive. For 23 of 31 OPV samples the sequence of the haemagglutinin (HA) gene had been determined by Sanger sequencing, revealing one ECTV and 22 CPXV infections. For eight OPV samples the HA sequence and therefore the OPV species were unknown at this point of time.

### Analysis of Multiplex Pyrograms

A cross-platform program MultiPSQ was developed in C++ using the Trolltech QT toolkit to perform the analysis of the pyrograms. Initially, the data created by the pyrosequencer was exported into an xml file using the Pyro-Mark ID System software. This file included information about the wells used in the experiment, the dispensation order and the times of injection of the dNTPs for each well as well as the raw signals recorded during the run. This xml file was loaded into the analysis software.

When reading the raw data of the wells, multiPSQ used a moving average filter to smooth the data. The baseline corrected value (B_i_) at each point was found by computing an intermediate value (A_i_) using the following formula:



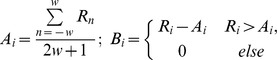



where R_n_ is the value of the raw data vector at position n, w is the window width (w = 30 in this case) and B_i_ is the value of the corrected vector at position i. Peaks were detected, with a peak being defined as a point which on the one hand was a local maximum, preceded by at least three monotonously rising points, followed by at least three monotonously falling points. On the other hand, it had to be close to a known dNTP dispensation time. Given that no significant signal decline was observed when analyzing fragments up to 30 flows in length, no correction for signal decline was performed. Once the data had been read and preprocessed, multiPSQ showed a list of used wells at the top and both raw and preprocessed data for the selected well at the bottom (Figure S1). The signals of either a single well or all wells could then be analyzed by clicking the “Analyze” or “Analyze all” buttons, respectively. The analysis was performed in five steps:

#### Calculation of possible pyrograms

MultiPSQ read the definition of the assay from a primer file and, given the dispensation order of the loaded experiment, calculated the pyrogram which would have been expected from each of the primers in each organism defined in the assay. The plain text primer file can be exported from the program mPSQed [13] for any multiplex pyrosequencing assay created therein or it can be created by hand following the instructions in the README file which is distributed together with multiPSQ.

#### Filtering of wells

To make sure that only wells containing a meaningful signal were analyzed, two criteria were defined which had to be fulfilled by the raw data from each well before it was considered for further analysis: A peak must have occurred at least once every four cycles (as only four different bases are dispensed periodically), and two consecutive peaks were not generated by an injection of the same base. If one of these anomalies occurred, the well was not considered in the following calculations and was classified as “suspected NTC” (non-template control).

#### Fitting of fingerprints

The pyrogram generated during the experiment could be seen as a vector of signal strengths which, if an organism defined in the assay was sequenced, must have been a linear combination of the pyrograms expected for that organism. Since the different sequencing primers could contribute differently to the final pyrogram, the formula for the expected fingerprint was:
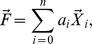
where the vector F is the fingerprint with each element of F representing the signal intensity following the dispensation of a base, n is the number of pyrosequencing primers in the assay, the vector X_i_ is the expected pyrogram of the i^th^ pyrosequencing primer with each element of X_i_ representing the expected signal intensity following the dispensation of a base given the dispensation order of the current experiment, and a_i_ is the factor with which the i^th^ pyrosequencing primer contributed to the fingerprint. Since neither the PCR amplification efficiencies nor the sequencing efficiencies of the different sequenced fragments relative to each other are known, it is impossible to pre-define a_i_ for any of the pyrosequencing primers. However, the values of F and X_i_ are known from the raw data and the assay definition file. Thus, as long as the number of dispensation events during the sequencing is larger than the number n of pyrosequencing primers used simultaneously in the experiment, the formula above describes an overdetermined system. Thus the set of factors a for each of the primers which allows the expected pyrograms to best fit the raw data could be calculated using the linear least squares method. Since a negative contribution of an amplicon to the signal is not biologically plausible, fingerprints for which the calculation yielded a negative value for any of the factors a_i_ were discarded at this point. This constraint additionally increases the power of the least squares method, facilitating the recovery of the correct factors a_i_ even in the presence of noise [14].

#### Filtering of fingerprints

If a peak was expected in the fingerprint but no peak could be detected in the raw data the fingerprint was discarded.

#### Determination of final classification

After the filtering step, the fingerprint which explained the peaks in the raw data with the highest R^2^ was defined as the most likely fingerprint. In order to assess the quality of this best fit, each assay can be calibrated using a plate with known species in each well. During calibration, the R^2^ of the best-fitting fingerprint for each of the wells is determined. A cutoff R_cut_ is defined as
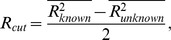
Where 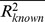 is the mean R^2^ of the best fit of the wells containing species defined in the assay and 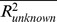 is the mean R^2^ of the best fit of the wells containing species not defined in the assay. In following analyses, any well where the R^2^ of the best-fitting fingerprint is lower than R_cut_ is classified as “unknown”. To ensure that the identified cutoff is correct, following calibration a leave-one-out analysis is performed over the whole plate. If, during the leave-one-out, any well is misclassified, the user is provided with a list of misclassified wells and warned that classification using the given assay is likely to be unreliable. Otherwise, the determined R_cut_ is saved to the assay definition file so that it can be used in subsequent analyses.

It is possible to perform classification without prior calibration. In that case, a default cutoff of 0.5 is used. However, every time an analysis is performed using an uncalibrated assay, the user is given a warning and is reminded to perform calibration to ensure valid results.

Since the algorithm only fits the raw signals to pyrograms expected from known, pre-defined sequences, a strain containing novel SNPs will be classified as “unknown”. The identification of sequences similar, but not identical to the predefined set is not possible since, as described above and shown in Figure 1, it is not possible to reconstruct the sequences from which a multiplex pyrosequencing fingerprint was created without a priori information about the possible starting sequences.

The results of the analysis for the selected well are shown in the top area of multiPSQ, along with the optimal factors a_i_ determined for the most likely fingerprint. For all wells, the analysis result is color-coded in the well selection area (Figure S1).

## Results

### Design of the Multiplex Pyrosequencing Assay

Critical steps in designing a multiplex pyrosequencing assay were the identification of suitable sequence variations and the respective selection of sequencing primers in combination with an optimal nucleotide dispensation order. Suitable SNPs could be identified by the following strategy: First, all relevant known sequences (sequences of 89 OPV genomes available from NCBI and 24 full genome sequences generated in-house which have not been published yet, see Table S1) were aligned. Then the sequences were grouped by species. Potentially interesting SNPs could be found at those positions in the alignment which are fully conserved within each of the individual groups but not fully conserved across the groups to be differentiated. Ideally, regions in the alignment should be identified which contain several such representative SNPs within a stretch of only a few bases, as several SNPs could then be sequenced with a single primer. Once characteristic SNPs had been found for each group, the pyrosequencing primers had to be positioned in a way that all of the SNPs could be sequenced with just a few sequencing cycles. Finally, the resulting fingerprints (Figure 1) had to be calculated to ensure that each group’s fingerprint was indeed unique. Because of the way the fingerprints were generated, it was possible for signals to mask each other. For instance, if one of the characteristic SNPs was the deletion of an A and another characteristic SNP – sequenced by a different primer – was the insertion of an A, these two SNPs would cancel out in the fingerprint if both “As” were sequenced in the same cycle. This problem could be solved by moving one of the primers so that one of them generates the sequence of the SNP in a different cycle. Obviously careful positioning of the pyrosequencing primers was of utmost importance. Specialized software had been developed in-house to aid in this design process [13].

The analysis and classification of pyrograms generated from such multiplex pyrosequencing assays was also challenging. Since software currently available was limited to a fixed number of SNPs and sequencing primers, we have developed our own software tool, multiPSQ, for data analysis. MultiPSQ was developed in C++ using the QT toolkit. Source code and binaries are available for download together with exemplary data from http://sourceforge.net/projects/multipsq/.

### Differentiation of Orthopoxviruses

**Figure 2:**
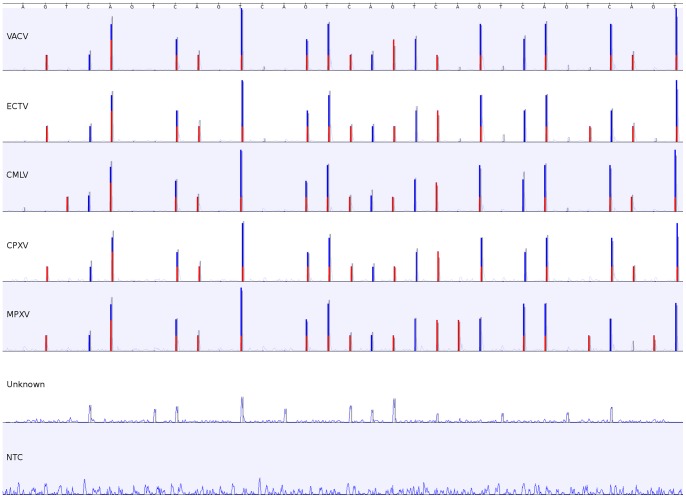
Fingerprints and species classification by the OPV multiplex PSQ assay. Each OPV species resulted in a slightly different fingerprint allowing a definite identification. Non-OPV template is determined as “unknown” and non-template control is identified as “NTC”.

In the case of OPV differentiation, pyrosequencing of one PCR product with two sequencing primers resulted in species-specific fingerprints for all OPV species tested (Figure 2). Samples with non-OPV template were correctly determined as “unknown” while the non-template control was truly negative. In addition to cell culture-derived virus stocks, 34 clinical samples from the German Consultant Laboratory for Poxviruses were analyzed with the new multiplex PSQ assay (Table 1). Pyrosequencing results were compared to conventional Sanger sequencing results, identifying 22 clinical samples as CPXV or ECTV positive, respectively, with both methods. Eight samples, for which no Sanger sequence was available at that time, were identified as CPXV positive using multiplex PSQ. In one case Sanger sequencing and pyrosequencing showed different results, classifying sample number 31 as CPXV or VACV positive, respectively. Examination of the regions used in the multiplex PSQ assay showed that sample 31 indeed carries all of the SNPs expected for VACV rather than for CPXV. However, phylogenetic analysis of the whole Sanger sequence showed sample 31 clearly clustering with CPXV. This means that the primers used in this work cannot be used to differentiate between CPXV and VACV since CPXV strains carrying the VACV SNPs exist. Still, the SNPs present in the sequence were correctly identified by the algorithm. Given that this work does not aim at creating a new diagnostic assay but rather at proving that relevant SNPs can be extracted from multiplex PSQ signals, and SNP information was indeed correctly identified, we consider this discrepancy in classification unproblematic.

**Table 1:** Comparative results of multiplex pyrosequencing and Sanger sequencing.

Sample #	Species	DNA source	OPV PCR	Sanger sequencing	Pyrosequencing
1	banded mongoose	skin	+	CPXV	CPXV
2	cat	scab	+	CPXV	CPXV
3	cat	swab	+	CPXV	CPXV
4	dog	tissue, paraffin	+	n.d.	CPXV
5	human	swab	+	CPXV	CPXV
6	human	scab	+	n.d.	CPXV
7	human	swab	+	CPXV	CPXV
8	human	swab	+	CPXV	CPXV
9	human	ichor	+	CPXV	CPXV
10	human	swab	+	CPXV	CPXV
11	human	skin	+	CPXV	CPXV
12	human	scab	+	n.d.	CPXV
13	human	scab	+	CPXV	CPXV
14	human	cornea	+	CPXV	CPXV
15	human	tissue	+	CPXV	CPXV
16	human	tissue, paraffin	+	n.d.	CPXV
17	human	tissue	+	n.d.	CPXV
18	human	swab	+	n.d.	CPXV
19	human	tissue	+	CPXV	CPXV
20	human	scab	+	CPXV	CPXV
21	human	swab	+	n.d.	CPXV
22	human	lymph node	+	CPXV	CPXV
23	human	glove	+	CPXV	CPXV
24	human	ichor	+	CPXV	CPXV
25	jaguarundi	skin	+	CPXV	CPXV
26	mara	eye	+	n.d.	CPXV
27	mouse	lung	+	ECTV	ECTV
28	rat	swab	+	CPXV	CPXV
29	rat	skin	+	CPXV	CPXV
30	rat	lung	+	CPXV	CPXV
31	rat	swab	+	CPXV	VACV
32	human	swab	−	PPV	−
33	human	serum	−	PPV	−
34	human	swab	−	MOCV	−
35		cell culture	+	CMLV	CMLV
36		cell culture	+	CPXV	CPXV
37		cell culture	+	CPXV	CPXV
38		cell culture	+	CPXV	CPXV
39		cell culture	+	ECTV	ECTV
40		cell culture	+	MPXV	MPXV
41		cell culture	+	VACV	VACV

+ positive PCR result; − negative PCR result; n.d. not done. Sanger classification is based on sequencing of a ∼960 bp fragment of the HA gene, except for PPV and MOCV which are not OPV (thus also yielding no signal in the OPV PCR).

Non-OPV species such as PPV and MOCV were identified as negative.

## Discussion

In contrast to laborious and time-consuming Sanger sequencing which is usually done to identify and type pathogens following PCR, pyrosequencing represents a fast and reliable alternative method. It allows sequence generation and pathogen identification in less than one hour from preparation of PCR products to final sequence. However, due to the underlying mechanism of sequencing-by-synthesis, sequences generated by pyrosequencing are usually short (up to 80 nucleotides) [6], [15]. Therefore, discrimination of more than two closely related organisms that differ only in a few but non-conserved SNPs is not possible with conventional pyrosequencing. To circumvent this problem, the generation of sequences starting from more than one primer leads to specific sequence fingerprints, enabling pathogen typing despite non-conserved SNPs that are representative for a respective group. Recently, such a multiplex pyrosequencing assay has been developed e.g. for genotyping hepatitis C virus [11].

Multiplex pyrosequencing is a versatile technology which can be used for quick and easy classification of samples based on a large set of different SNPs. The design of assays and the analysis of the resulting data are, however, neither trivial nor are they supported by common software. In this paper, we present the program MultiPSQ which complements the analysis capabilities of the Qiagen pyrosequencing platform. We demonstrate the capability of this technology to tackle non-trivial tasks by applying it to the identification and classification of all human-pathogenic OPV.

In our investigations one clinical sample could not be classified correctly by the novel approach. The multiplex assay was designed on the basis of all 89 known and annotated OPV genomes found in GenBank (see Table S1) and 24 unpublished OPV genomes sequenced by whole genome sequencing in our lab. Sanger sequencing of the longer haemagglutinin ORF (816–957 bases) identified a CPXV strain in clinical sample number 31. However, the CPXV strain shows sequence similarity to VACV strains in the region of the O2L CDS used for multiplex pyrosequencing. The use of a third amplicon for pyrosequencing could help to obtain additional information.

Since our method classifies samples based on the similarity of their pyrosequencing fingerprints to theoretical fingerprints generated from known sequences, novel mutations cannot be correctly attributed to a certain species, but can be defined as unknown, indicating a novel variant of the virus. As can be seen in Figure 1, it is impossible to reconstruct the individual sequences from the fingerprint. Thus, any unknown strain – in this case for instance an OPV with a novel mutation in the sequence regions used – will generate a fingerprint which is different from all of the expected fingerprints. Since it is impossible to tell whether this difference stems from a simple SNP or from major differences in sequence, the sample will be classified as “Unknown”.

It has to be mentioned that a multiplex pyrosequencing assay is not suitable to identify co-infections with different pathogen strains. We have demonstrated the applicability of multiplex pyrosequencing to the identification and classification of human-pathogenic OPV. This method is applicable to any problem where samples need to be classified based on nucleic acid sequences. The possibility to use any number of amplicons with any number of pyrosequencing primers and thus any number of SNPs means that even tough problems, where conserved sites with discriminating SNPs are few and far between, can be successfully tackled. However, since our method does not correct for the decline of signal intensity in long pyrosequencing reads, it may not perform well on assays in which long amplicons need to be sequenced.

We postulate that the method presented in this paper will lead to a more wide-spread adaptation of multiplex pyrosequencing in diagnostics, as it allows the quick and cost-effective generation of high-quality results.

## Supporting Information

Figure S1 **Screenshots of the program.** Left: The program window after a run xml file has been loaded into the software. A: The imported plate is shown, the used wells are marked. The raw data (B: gray graph) and the pre-processed data (C: blue graph) are displayed. The gray bars in the blue graph show where peaks have been detected. Right: The program window after a run has been analyzed. D: Next to the plate graphic the resulting classification (in this case CPXV1) and the optimum factors of the (two) pyrosequencing primers are shown. E: Red and blue bars in the bottom graph visualize how the detected peaks are explained by the signals expected from the two primers.
(TIFF)

Table S1 List of 89 OPV genomes from NCBI used in the design of the proof-of-concept assay.
(XLS)
