# Supplementary figures and images for: MultiPSQ: A Software Solution for the Analysis of Diagnostic n-Plexed Pyrosequencing Reactions
Source: PLoS One. 2013 Mar 26;8(3):e60055. doi: 10.1371/journal.pone.0060055 (PMC3608623; doi:10.1371/journal.pone.0060055)

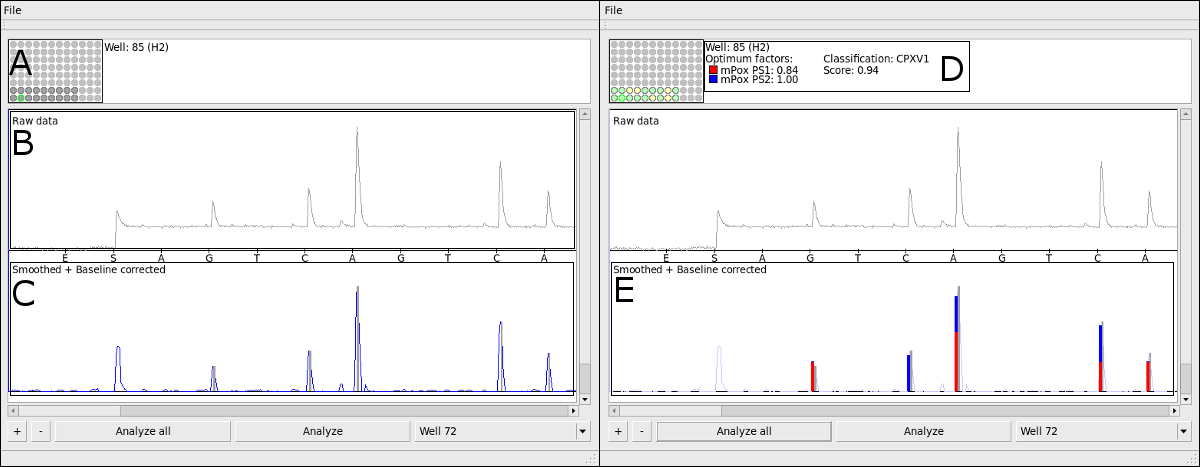

Supplement: Figure S1 — Screenshots of the program. Left: The program window after a run xml file has been loaded into the software. A: The imported plate is shown, the used wells are marked. The raw data (B: gray graph) and the pre-processed data (C: blue graph) are displayed. The gray bars in the blue graph show where peaks have been detected. Right: The program window after a run has been analyzed. D: Next to the plate graphic the resulting classification (in this case CPXV1) and the optimum factors of the (two) pyrosequencing primers are shown. E: Red and blue bars in the bottom graph visualize how the detected peaks are explained by the signals expected from the two primers. (TIFF) [file pone.0060055.s001.tiff]
